# Quality of Life and Family Support in Critically Ill Patients following ICU Discharge

**DOI:** 10.3390/healthcare11081106

**Published:** 2023-04-12

**Authors:** Konstantina Avgeri, Epaminondas Zakynthinos, Vasiliki Tsolaki, Markos Sgantzos, George Fotakopoulos, Demosthenes Makris

**Affiliations:** Department of Medical School, University of Thessaly, 41110 Larissa, Greece

**Keywords:** ICU, patients’ support, family support, PICS, quality of life, critical care

## Abstract

Background: Following discharge from the intensive care unit (ICU), critically ill patients may present cognitive dysfunction and physical disability. Objectives: To investigate the quality of life (QoL) of patients following discharge from ICU, physical performance and lung function and to assess the role of support by family members and friends. Methods: This prospective study was conducted in the University Hospital of Larissa Greece between 2020 and 2021. Patients hospitalized at the ICU for at least 48 h were included and assessed at hospital discharge, at 3 and at 12 months later. The research implements of the study were a dedicated questionnaire and the SF-36 health questionnaire for the appraisal of the QoL. Lung function changes were assessed by spirometry and physical performance by the 6-min walking test (6MWT). Results: One hundred and forty-three participants were included in the study. The mean (SD) of the physical and mental health SF-36 scores at hospital discharge, 3 and 12 months were 27.32 (19.59), 40.97 (26.34) and 50.78 (28.26) (*p* < 0.0001) and 42.93 (17.00), 55.19 (23.04) and 62.24 (23.66), (*p* < 0.0001), respectively. The forced expiratory volume in one second and 6MWT significantly improved over 12 months. Patients who were supported by two or more family members or patients who were visited by their friends >3 times/week presented better scores in the physical and mental SF36 domains at 12 months. Conclusion: This study shows that the quality of life of Greek patients who were discharged from the ICU can be positively affected both by the support they receive from their family environment and friends.

## 1. Introduction

Intensive care unit (ICU) hospitalization is a considerably stressful situation both for patients and their families [1,2] and has various physical and mental implications. Patients following ICU management may present reduced lung function [3], neuromuscular dysfunction, anxiety and depression. Six months following ICU discharge, patients showed reduced functional and pulmonary capacity, while an improvement was observed at 12 months after discharge [3]. These disorders in physical, intellectual and mental health have been described as post-ICU traumatic syndrome [1] and may be present for years, compromising the QoL of patients [4,5]. Family members may have an important role in the management of a critical illness, because they are often responsible for making decisions that the patients are unable to make on their own. Studies show that more than 50% of patients have to be taken care of by family members [6,7]. In this respect, family members’ support is pivotal in improving the patients’ health by contributing to quality care [8]. In turn, this has an impact on the lives of those family members. Indeed, when a patient is at the ICU in critical condition, family members may also suffer from symptoms such as anxiety, acute stress disorder, post-traumatic stress disorder, depression and complicated grief [9]. In this respect, the long-term impact of a critical disease on the QoL of both patients and family and the role of family in supporting critical care patients are important for planning effective supportive healthcare networks. Nevertheless, data regarding the impact of family support on ICU patients’ post-ICU, especially in Greece, are limited.

In this study we aimed to investigate the QoL of ICU patients after their discharge from a Greek ICU and to evaluate the impact of family on their QoL over a one-year period. Furthermore, we aimed to assess lung functional changes and physical performance over this period.

## 2. Methods

This was a prospective study conducted in a tertiary hospital in Larissa, Greece, between 2020 and 2021. Patients were included if they (a) were discharged from the ICU following >48 h hospitalization and (b) were able to perform spirometry and the six-minute walking test (6MWT) at hospital discharge based on treating physicians’ decisions and agreed to complete a questionnaire assessing the QoL and support received in daily activities.

The study was approved by the local ethics committee of the University Hospital of Larissa (No. 43704). Informed consent was obtained by the patient or next of kin.

### 2.1. Study Outcomes

The relationship between the overall SF-36 score at 12 months following hospital discharge and the support in daily activities (hours/day) received by family members and friends in total was the primary outcome in the study. Secondarily, we assessed the exercise performance by the 6MWT and lung function by spirometry.

### 2.2. Data Collection

Participants were evaluated at hospital discharge at 3 and 12 months. Patient medical records were evaluated to obtain demographic data, the severity of critical illness by the Acute Physiology and Chronic Health Evaluation (APACHE II) score, cause of admission, length of ICU stay, medical problems, medications and QoL variables.

The criteria for ICU admission/discharge or hospital discharge were left to the discretion of the treating physicians.

### 2.3. Questionnaire Interview

A dedicated questionnaire and an SF-36 questionnaire were implemented to assess the role of family support and the QoL. The questionnaire included items assessing the support received by spouses, family and friends based on the previous literature [6,10]. Data were adjusted for segregated care hours. Specifically, support received by spouses/family was classified as 1–4 h daily, 4–8 h daily and 24 h daily. Support received by friends was classified arbitrarily as every day, 3–4 times/week, once/week, 1–2 times/month and never. The SF-36 questionnaire includes multi-item scales measuring each of eight generic health concepts: physical functioning (PF), role limitations due to physical health problems (RP), bodily pain (BP), general health perceptions (GH), vitality (VT) tapping energy levels and fatigue, social functioning (SF), role limitations due to emotional problems (RE) and mental health (MH). Each item is weighted with an additive scaling to calculate the final domain score. A high score indicates a low impairment, and a low score designates an important impairment. The questionnaire is valid for the Greek population. In the present study, we arbitrarily used the median scores of participants in the physical and mental components of the questionnaire to classify patients as those with improved scores (≥ median) and those with deteriorating scores (< median). Completion of the questionnaires was not during the scheduled interviews. The schedule was carried out by telephone communication. The questionnaires were completed in person by participants in outpatient clinics. In cases where the presence of the participant in the hospital was not possible, the evaluation was performed at home. In cases where the participant was not able to complete the questionnaire alone, the questions were answered with the assistance of the next of kin.

### 2.4. Respiratory Function Assessment

Lung function tests included spirometry to assess the forced expiratory volume in one second, (FEV1) and forced vital capacity of the lungs (FVC). Spirometry was performed at baseline and at the end of each time period with a computerized system. This system, which meets the ATS standards, was calibrated every day with standardized techniques according to the guidelines [11]. Pulse oximetric saturation (SpO2) was recorded immediately before each measurement using pulse oximetry (Nonin 8500 M; Nonin Medical; Minneapolis, MN, USA).

### 2.5. 6-Min Walk (6MWT)

The 6MWT was performed indoors about the same time of day along a 100-foot flat, straight, enclosed hallway with a hard surface that was seldom traveled. The walking course was 30 m in length, and it was marked every 3 m. Instructions to patients were given according to the accepted recommendations. The patient should sit at rest in a chair located near the starting position for at least 10 min before the test started. Clothing and shoes should be appropriate for walking. During that time, oxygen saturation, pulse and blood pressure were measured and baseline dyspnea was assessed using the Borg scale. A physician should stand near the starting line during the test without walking with the patient. Only the standardized phrases for encouragement were used during the test. When the test was finished, the post-walk Borg dyspnea, oxygen saturation and pulse rate were recorded, as well as the total distance covered [12].

### 2.6. Statistical Analysis

Data are expressed as mean (standard deviation (SD)) or median ((interquartile range (IQR)) or n (%). Normality was assessed by the Shapiro–Wilcoxon test. Comparisons between patients were performed using a Mann–Whitney test for continuous variables by *t*-test and nonparametric test. The means of two or more independent groups were compared by one-way ANOVA. All statistical tests were 2-sided. A result was considered statistically significant when *p* < 0.05. Analyses were performed using the SPSS v.25 software (ILLINOIS, USA).

## 3. Results

**Figure 1:**
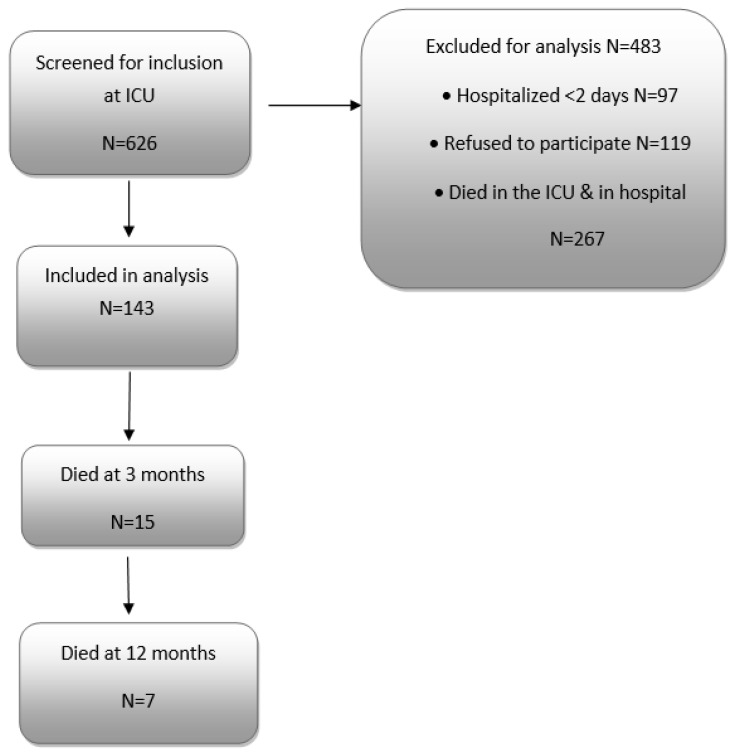
Flow chart of the study.

Overall, 143 patients were included in the study (Figure 1). Sociodemographic characteristics and baseline clinical characteristics of participants are shown in Table 1 and Table 2, respectively.

**Figure 2:**
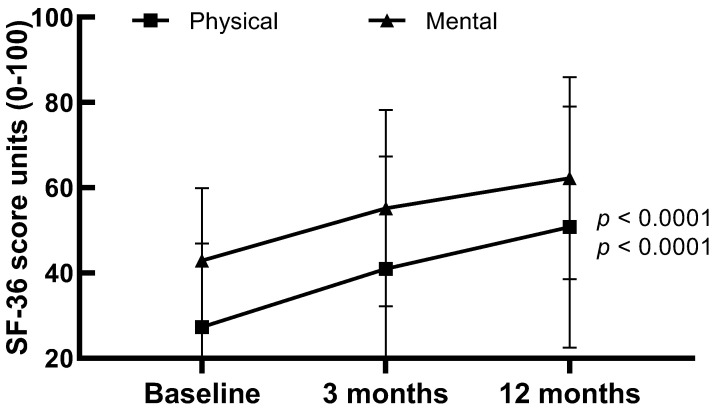
Physical and mental health scores of participants at different time points following ICU discharge. Data are presented as mean (SD) values.

Figure 2 presents the SF-36 physical and mental health component scores of the participants over 12 months following their hospital discharge. The SF-36 physical scores at hospital discharge, 3 months and 12 months were 27.32 (19.59), 40.97 (26.34) and 50.78 (28.26) (*p* < 0.0001), respectively; the mental health scores at hospital discharge, 3 months and 12 months were 42.93 (17.00), 55.19 (23.04) and 62.24 (23.66) *p* < 0.0001), respectively.

### 3.1. Lung Function and 6MWT

**Figure 3:**
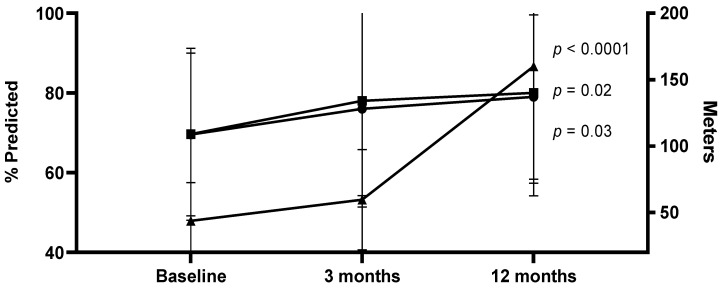
Spirometric values (forced expiratory volume in one second (FEV1) and forced vital capacity (FVC) and the six-minute walking test (6MWT) distances at different time points following ICU discharge. Data are presented as mean (SD) values.

Lung function in terms of spirometric values and patients’ performances in the 6MWT over time are presented in Figure 3 The 6MWT distances (meters) at discharge, 3 months and 12 months were 43.8 (28.8), 59.6 (37.8) and 160.4 (97.5) (*p* = 0.0001), respectively.

The forced expiratory volumes in one second (FEV1) (liters) were 2.20 (0.81), 2.30 (0.92) and 2.40 (0.81), (*p* = 0.013), respectively, and the forced vital capacity (FVC) (liters) was 2.63 (0.96), 2.80 (1.11) and 2.95 (0.93), (*p* = 0.023), respectively. 

### 3.2. Family Support and SF-36 Scores

**Figure 4:**
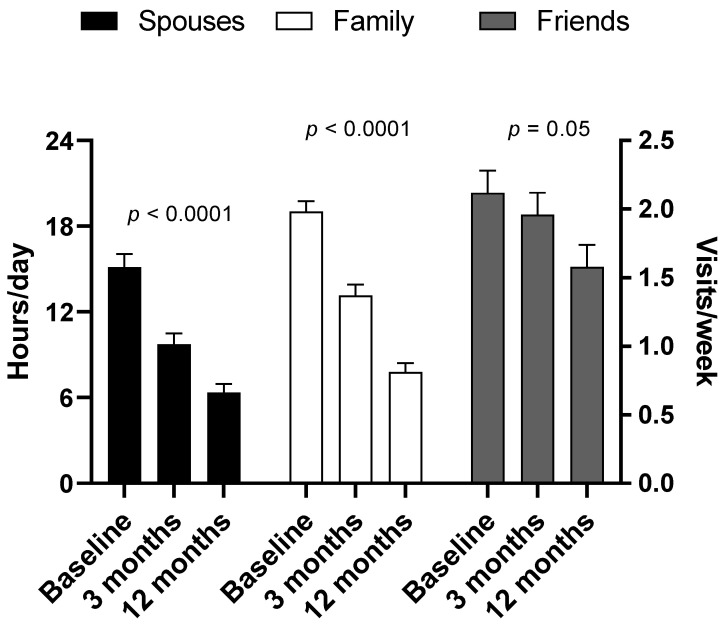
Duration of daily support by family members/spouses (hours/day) and frequency of visits by friends (number of visits/week) to patients at different time points following ICU discharge. Data are presented as mean (SD) values.

Figure 4 presents details on daily support at different time points following hospital discharge. One-hundred and forty-two out of one-hundred and forty-three (99.30%) patients received support by one or more family members, one hundred and thirteen (79.02%) by their spouses and eighty-two (42.65%) by one or more friends. Patients received support in their daily activities by their spouses at baseline, 3 months and 12 months for 15.14 h/day, 9.76 h/day and 6.35 h/day (*p* < 0.0001), respectively.

Male patients received significantly more frequently support by their family in terms of hours/day compared to females (*p* = 0.03). There was no association between the gender and the number of family members involved in either supportive care or with the duration of support by spouses or with the number of visits by friends. Participants with basic education received significantly more frequent support by their family in terms of hours/day. No other significant association was found between the demographic factors and types of support by family, spouses and friends.

Table 3 and Table 4 present patients with improved SF-36 scores (≥median of the relevant score of the total population or not) at 12 months follow-up. Patients with ≥median scores had significantly increased lung function compared to patients who presented lower than the median score. Patients with ≥median scores presented also shorter ICU stays (*p* = 0.0004) and hospital stays (*p* = 0.014). Those who presented ≥median SF-36 scores at 12 months also had significantly lower frequencies of a stroke at admission (*p* = 0.001). Participants with ≥median scores were supported more frequently by more than two family members daily or by their friends (more than three times per week).

## 4. Discussion

The main findings of the present study are (a) participants who received more frequent care by more than two members of their families presented better QoL at 3 and 12 months after their discharge from hospital compared to patients who received care by fewer members, (b) participants with ≥median of the SF36 score of the total population at 12 months were supported more frequently by more than two family members daily or by their friends (more than three times per week) compared to patients with <median scores and, (c) similarly, participants with ≥median of the SF36 score of the total population at 12 months had higher values in spirometry or in the 6MWT compared to patients with <median values of the cohort.

The evidence shows that, even years after ICU admission, patients’ QoL are significantly decreased compared to the general healthy population [13]. According to Wytske et al. (2021) [14], patients presented several problems both physically and cognitively one year after being admitted to the ICU. The present study assessed the QoL during a 12-month period at three different time points following ICU discharge in a Mediterranean region during the COVID-19 pandemic. Our results suggest that patients recovered gradually in terms of their QoL and presented maximum improvement in their SF-36 scores at twelve months. The SF-36 scores in the physical and mental domains were 45.5 (29.6) and 57.8 (24.7), respectively; these values were significantly higher compared to the respective values at hospital discharge and at the 3-month follow-up. There are no available data for the SF-36 score evolution over time in this setting in Greece. The mean SF score in a population with heart disease in Sweden was 70 [15]. In this respect, one might argue that our population, despite the improvement in QoL at 12 months following ICU, still had compromised QoL at that time point.

Previous studies of the field have suggested that the majority of long-term care for adult patients, either at home or in a community facility, is provided by 90% of their family members [16,17]. The present study shows that patients who were supported more frequently by two or more family members had SF-36 scores at 12 months that were ≥median value of the total cohort compared to patients who were less supported. A plausible explanation might be that the quality of care may be enhanced when many family members are involved in supporting a patient. We speculate that it is possible to provide better help in practical issues (i.e., patients’ mobility, enhanced communication and household help) or they may offer psychological support, both important for patients to recover better and faster. Another plausible explanation might be that aged persons have fewer social networks, and therefore, the difference in QoL may be due to age and associated comorbid conditions rather than the presence of social support itself. We believe that a future investigation could define if a specific type of support may be significantly associated with recovery.

Previous studies showed that the relationship between spouses’ care and the course of patients’ health is important. Spouses may spend long hours every day transferring and helping their spouses who cannot care for themselves. In almost two-thirds of critically ill patients, it is their spouses who cared for them after their discharge from the ICU. Furthermore, younger spouses and females played a more active and regular role in the care of patients compared to elderly or male ones [10,18,19]. In this study, we found that ICU patients present better QoL when their spouses cared for them for more than 8 h daily. In Greece, there are certain deficiencies in the organized distributed support [20] by the state to seriously ill patients when they return to their home environment. It would be of benefit for patients following their discharge from the ICU and hospital to be supported by specialized groups of professionals that can provide home care and can assist spouses and other siblings who live with the critically ill patients.

In this study, we found that the shorter the stay in the ICU for a patient, the better their QoL will be. Previous studies of the field have shown an adverse association between the length of stay in the ICU and the QoL of patients. Notably, when mechanical ventilation (MV) was used for more than seven days, patients manifested worse QoL. In addition, staying in the ICU for more than ten days was associated with higher mortality rates [18]. The contribution of the family is very important in this case as well. Family members’ support and visits at the ICU can reduce the length of stay; it has been observed that communication and showing deep love and affection may affect the QoL positively [6,21,22]. In this respect, ICU planning should incorporate the maximum possible support from relatives, when possible, to maximize their benefits and facilitate patients’ recovery.

In addition, we found that QoL was associated with the presence of acute neurological illness. Previous studies have shown that stroke is the second leading cause of death and disability worldwide, and depending on the severity of the stroke, it can cause reduced physical fitness and quality of life. The consequences for the reduced QoL of patients suffering from a stroke are related to the duration of stay at the hospital and the program that they followed when discharged [23,24,25,26]. These patients cannot be independent, because most of them have permanent disabilities and must change their daily lives. In this respect, they may need support from their family environment for their self-care. Needless to say, the contribution of a specialized staff to help them deal with their problems and improve their health is of crucial importance.

In the present investigation, we assessed patients’ physical performances and lung function following the ICU in terms of the 6MWT distance and spirometry. Previous studies have suggested that ICU patients who survived four months after discharge had significantly worse outcomes than the healthy population [27,28]. The 6MWT performance of ICU patients has been studied in severe respiratory disease; ARDS survivors presented significantly reduced 6-min walking distances at six months and one year follow-up [29,30,31]. In our study, we found improvement in the 6MWT distance at 3 and 12 months after ICU discharge, however, the absolute distances were lower compared to healthy patients who usually present higher 6MWT distances (over 600 m) [28]. It remains elusive whether ICU patients may regain their previous performances over longer time periods. Similar to the 6MWT distance, patients’ lung function presented significant improvement over 12 months in our study. Previous investigations showed that patients with ARDS presented mild abnormalities in lung spirometry following the ICU [32,33,34] or they may have presented fluctuations that were within the normal limits during 3 to 5 years of follow-up.

The present study presents certain limitations that should be taken into consideration when interpreting its findings. First, this is a one-center study that presents data from a specific area in Central Greece, and the sample size of the population studied may be relatively small to evaluate specific subgroups. However, this center provides services to a respectively large population of people, and the results of the study could be useful in implementing strategies at the local level. Moreover, the questionnaire used in the study did not include questions with details on patients’ pre-hospital stay or their specific mental support following patients’ discharge from hospital. In addition, this study does not provide details related to public and private health support for the patients. This type of support is not standard, and thus, we cannot evaluate its impact on the QoL of patients. Furthermore, the study does not provide details for MV variables in terms of the MV mode, the MV settings used in the ICU or the mechanical properties of the respiratory systems of the participants. We certainly acknowledge that these details may have provided more insight on the evolution of patients’ health over time.

## 5. Conclusions

In conclusion, this observational study suggests that critical care patients presented significant improvement at 12 months following ICU admission in their QoL, lung function and physical performance in terms of the SF-36 assessment, spirometry and 6MWT, respectively. Daily support by family members and frequent visits by friends may have a positive impact on the QoL of critical care patients following their discharge from the hospital.

## 6. Relevance to Clinical Practice

The role of family members and friends is particularly important for patients after their discharge from the ICU. Family members and friends support during patients’ daily activities may help in their recovery and improved quality of life in the long term. The participation of family members could be incorporated into relevant programs that aim to improve patients’ recovery from critical illnesses.

## Figures and Tables

**Table 1:** Social and demographic characteristics of participants.

Age, years	56.8 (17.49)
Female Sex, n (%)	57 (39.86)
Family Status	
Married, n (%)	106 (74.12)
Not-married, n (%)	31 (21.68)
Widow/er, n (%)	6 (4.20)
Accommodation	
Urban, n (%)	76 (56.70)
Daily area, n (%)	34 (25.40)
Agricultural, n (%)	24 (17.90)
Education	
Basic education, n (%)	28 (19.59)
High School graduates, n (%)	86 (60.15)
Technical School graduates, n (%)	6 (4.19)
University graduates, n (%)	10 (6.99)
Post graduate training, n (%)	3 (2.09)
No education, n (%)	10 (6.99)
Type of Profession	
State employee, n (%)	13 (9.09)
Private employee, n (%)	25 (17.5)
Freelance, n (%)	9 (6.30)
Farmer, n (%)	10 (6.9)
Worker, n (%)	6 (4.19)
Housekeeper, n (%)	14 (9.80)
Retired, n (%)	52 (36.36)
University student, n (%)	5 (3.49)
Unemployed, n (%)	9 (6.29)
Rehabilitation after ICU, n (%)	70 (48.9)
Time to return to daily routine after ICU	
1–6 months, n (%)	58 (40.5)
6–12 months, n (%)	31 (21.7)
>12 months, n (%)	54 (37.3)

Data are presented as the mean (SD) unless otherwise indicated.

**Table 2:** Clinical characteristics of participants in the study.

Cause of Admission	
-Medical, n (%)	79 (55.25)
-Pneumonia, n (%)	6 (4.20)
-ARDS, n (%)	10 (6.99)
-Stroke, n (%)	31 (21.6)
Surgical, n (%)	48 (33.56)
APACHE II score	19 (1.1)
Mechanical ventilation, n (%)	126 (88.1)
Mechanical ventilation duration, (median (IQR)), days	5 (2–12)
ICU stay, (median (IQR)), days	7 (3–14)
Hospital Stay, (median (IQR)), days	20 (15–20)
Spirometry *	
FEV1, %pred	69.5 (21.5)
FVC, %pred	69.5 (20.4)
FEVI/FVC, %pred	106 (97–121)
PEF, %pred	58.5 (25.1)
6MWT *, meters	43.84 (28.79)

Data are presented as the mean (SD) unless otherwise indicated. * Values at hospital discharge. ARDS: acute respiratory distress syndrome; FEV1: forced expiratory volume; FVC: forced vital capacity; FEV1/FVC: the ratio of the forced expiratory volume in the first second compared to the forced vital capacity of the lungs; PEF: peak expiratory flow; 6MWT: six-minute walking test.

**Table 3:** Participant characteristics according to the median value of the physical domain of the SF36 questionnaire at 12 months.

	≥Median SF36 Score N = 71	<Median SF36 Score N = 72	*p* Value
Age, years	54 (35–69)	66 (52–75)	0.0001
Male, n (%)	43 (60.5)	42 (58.3)	0.6
Stroke, n (%)	7 (9.8)	24 (33.3)	0.0001
ICU stay > 10 days, n (%)	17 (23.9)	36 (50)	0.001
Hospital stay > 10 days, n (%)	58 (81.6)	69 (95.8)	0.001
FEV1, % pred	86 (73–97.7)	49.5 (43.2–54.7)	0.0001
FVC, % pred	81 (68–93)	48 (44–53.5)	0.0001
FEV1/FVC, % pred	104 (95.5–112.5)	46 (43–52)	0.0001
PEF, % pred	81 (69–95)	47 (38–51)	0.0001
6MWT, meters	153 (99–250)	65 (38–101.5)	0.0001
Support by spouses 24/24 h, n (%)	10 (14.0)	12 (16.6)	0.8
Friends’ visits > 3/week, n (%)	39 (54.9)	14 (19.4)	0.0001
Family-Support > 2 members, n (%)	61 (85.9)	46 (63.8)	0.001

Data are presented as the median (IQR) unless otherwise indicated. FEV1: forced expiratory volume; FVC: forced vital capacity; FEV1/FVC: the ratio of the forced expiratory volume in the first second compared to the forced vital capacity of the lungs; PEF: peak expiratory flow; 6MWT: the six-minute walking test.

**Table 4:** Participant characteristics according to the median value of the mental domain of the SF36 questionnaire at 12 months.

	≥Median SF36 Score N = 73	<Median SF36 Score N = 70	*p* Value
Age, years	54 (36.5–69)	65.5 (53–74)	0.0001
Male, n (%)	45 (61.6)	40 (57.1)	0.9
Stroke, n (%)	7 (9.6)	24 (34.3)	0.001
ICU stay > 10 days, n (%)	19 (26)	39 (55.7)	0.001
Hospital stay > 10 days, n (%)	57 (78)	69 (97.1)	0.002
FEV1, % pred	88 (74–100)	54 (48.5–58.5)	0.0001
FVC, % pred	84 (78–99)	60 (52–65)	0.0001
FEV1/FVC, % pred	104 (95.5–112.5)	46 (43–52)	0.0001
PEF, % pred	88 (78–97)	49 (39.5–55)	0.0001
6MWT, meters	170 (106–250)	36.6 (7.5–58)	0.0001
Support by spouses 24/24 h, n (%)	12 (16.4)	10 (14.3)	0.3
Friends’ visits > 3/week, n (%)	36 (49.3)	17 (24.3)	0.001
Family-Support > 2 members, n (%)	60 (82.2)	47 (67.1)	0.003

Data are presented as the median (IQR) unless otherwise indicated. FEV1: forced expiratory volume; FVC: forced vital capacity; FEV1/FVC: the ratio of the forced expiratory volume in the first second compared to the forced vital capacity of the lungs; PEF: peak expiratory flow; 6MWT: the six-minute walking test.

## Data Availability

Any data related to the study can be provided upon a reasonable request.
